# Evaluating the Realism of a Task Trainer and the Impact on Residents’ Confidence in Managing Massive Upper Gastrointestinal Bleeding Among Emergency Medicine Residents

**DOI:** 10.7759/cureus.73076

**Published:** 2024-11-05

**Authors:** Amrita Vempati, Ashley Tarchione

**Affiliations:** 1 Emergency Department, Valleywise Health Medical Center, Phoenix, USA; 2 Emergency Medicine Department, Creighton University School of Medicine, Phoenix, USA; 3 Emergency Medicine Department, Kaiser Permanente, San Diego, USA

**Keywords:** constructing low-cost relatively high-fidelity task trainers, emergency medicine procedural competency, minnesota tube, simulation realism, upper gastrointestinal bleed

## Abstract

Objective

Hemodynamically unstable upper gastrointestinal bleeding (UGIB) represents a life-threatening emergency that often lacks adequate high-fidelity training in the insertion of balloon tamponade devices. To address this gap, we developed an affordable task trainer that provides real-time feedback to enhance the training experience for emergency medicine residents. This study aims to evaluate the realism of the task trainer and its impact on residents' confidence in managing massive UGIB.

Methods

Constructed from low-cost, readily available materials, the task trainer was utilized in a training session attended by 28 emergency medicine residents. Following the review of an instructional video, participants were timed during the insertion of the balloon tamponade device. Subsequently, they completed a paper survey to assess their experiences and confidence levels.

Results

All 28 residents completed both the pre- and post-session surveys. The average time for inserting the balloon tamponade device was 3 minutes and 49 seconds. Sixteen participants strongly agreed, 11 agreed, and one felt neutral regarding the task trainer's realism in teaching the procedure. Additionally, all residents rated the simulation average or above average compared to previous educational experiences. Fourteen residents reported feeling significantly more confident in managing massive UGIB, while 13 felt slightly more confident, and one noted no change in confidence. Participants provided several positive remarks regarding the training session.

Conclusions

The balloon tamponade device task trainer proved beneficial in enhancing emergency medicine residents' confidence in managing patients with massive UGIB. Furthermore, the task trainer was perceived as realistic and superior to other educational modalities.

## Introduction

Upper gastrointestinal bleeding (UGIB) can be categorized into non-variceal and variceal causes. Non-variceal UGIB is typically due to conditions such as peptic ulcer disease, mucosal tears, or vascular malformations, whereas variceal UGIB primarily results from gastroesophageal varices secondary to liver cirrhosis [1]. Massive UGIB is characterized by any of the following: 1) hemodynamic instability; 2) signs of poor perfusion (e.g., altered mental status, syncope, pallor); 3) the need for more than two units of packed red blood cells during initial resuscitation; or 4) rapid and overt bleeding [2]. Varices account for approximately 14% of massive UGIB cases, making them the second most common cause [3]. The mortality rate for variceal massive UGIB can reach up to 60% [4].

Patients with massive UGIB who present to the emergency department are at risk of rapid deterioration and require immediate interventions such as airway management, fluid resuscitation with crystalloids, blood transfusions, and medication administration [3]. In severe cases, balloon tamponade devices like the Minnesota or Sengstaken-Blakemore tubes may be used to control bleeding. Among patients who receive balloon tamponade, 59% survive hospital discharge, and 41% are alive after one year [5]. As the initial providers responsible for managing these critical situations, emergency medicine physicians must be trained in all aspects of UGIB management.

Although the insertion of balloon tamponade devices is not included in the Accreditation Council for Graduate Medical Education's core procedures for emergency medicine training [6], the American Board of Emergency Medicine's Model of Clinical Practice specifies "mechanical control of upper gastrointestinal bleeding" as a required competency for emergency medicine residents [7]. Thus, training residents in the use of balloon tamponade devices is essential.

A variety of educational approaches are used to train emergency medicine residents, including classroom lectures, whiteboard teaching, and simulation. Simulation-based education, in particular, has become increasingly popular for procedural training because it provides learners with hands-on experience [8]. Task trainers are commonly employed for procedural skills training, offering varying levels of realism [9]. For the management of UGIB, there are currently videos, articles, and simulation scenarios available for teaching [4,10-11]. Although some task trainers for balloon tamponade device placement exist, most lack the realism necessary to provide meaningful feedback to learners during the procedure [12,13]. To address this gap, we developed a task trainer that is effective for teaching the procedure, supports repetitive and deliberate practice, and is easily reproducible.

This study aims to achieve two primary objectives: assessing the realism of the task trainer and enhancing the confidence of emergency medicine residents in managing massive UGIB.

## Materials and methods

Model construction

**Figure 1:**
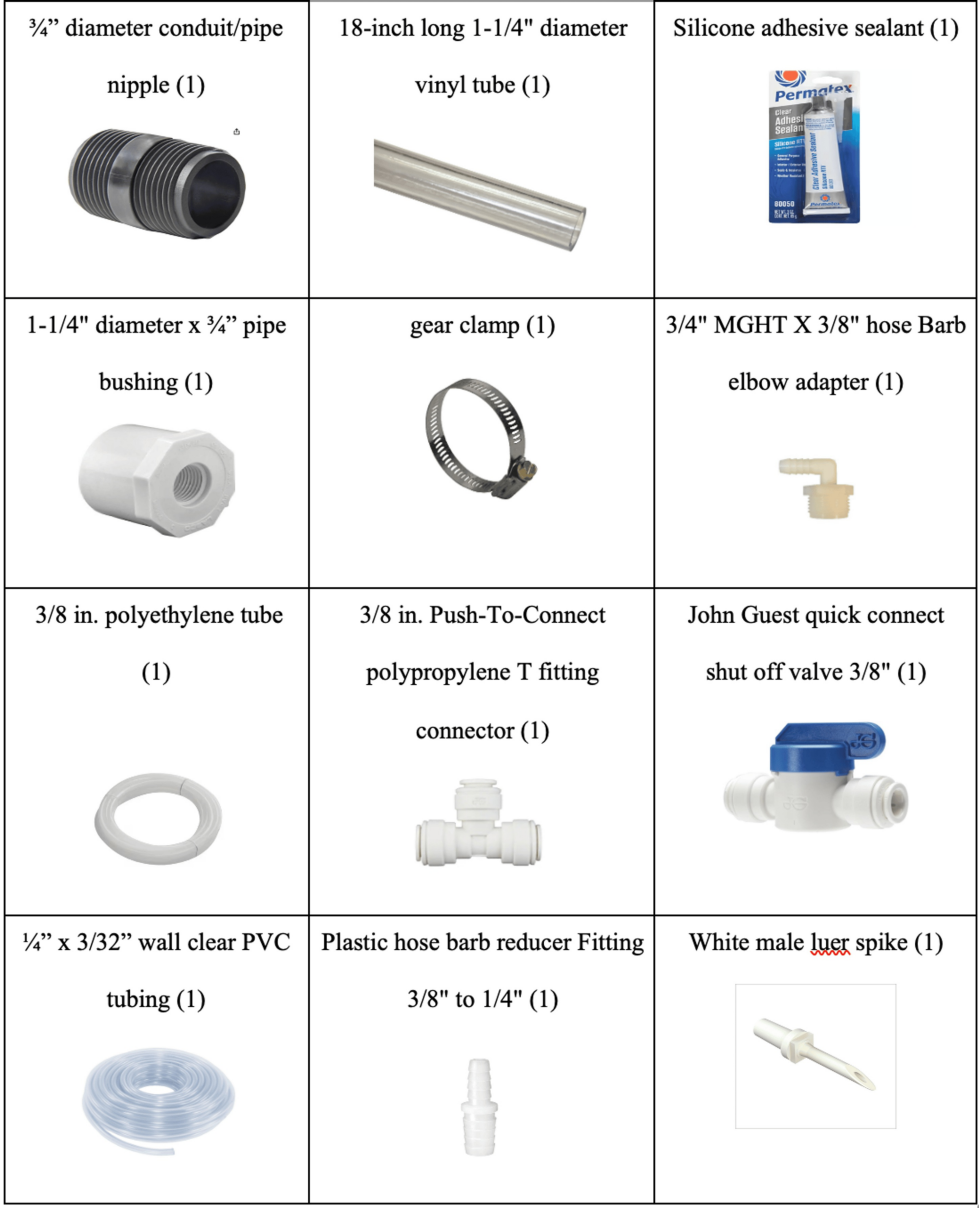
Parts list with pictures

Figure 1 contains the parts list for the task trainer construction. The total cost of the materials was about $40. All the parts are available either online or at a local home improvement store. The intravenous tubing, white male luer spike, and intravenous pole are available at a simulation center.

We used one of our Noelle^Ⓡ^ manikins to build the task trainer. The Noelle^Ⓡ^ manikin has both a trachea and an esophagus, which ends in a blind pouch. We also built this task trainer on our Laerdal^Ⓡ^ airway manikin.

*Task Trainer Construction Steps*

Step 1: Attach items to the blind esophagus: Cut through the esophagus of manikin that often ends in a blind pouch and begin by taking a ¾” diameter pipe nipple. Insert the pipe nipple through the designated opening in the blind esophagus section of your task trainer model. Secure the pipe nipple in place using a gear clamp. Ensure it is tightly fastened to prevent any movement or leakage.

Step 2: Attach the vinyl tube to the esophagus: Take an 18-inch long section of 1¼” diameter vinyl tube. Position one end of the vinyl tube onto the exposed end of the pipe nipple from Step 1. Secure the connection using another gear clamp, ensuring a snug fit to prevent any disconnections during use.

Step 3: Insert the pipe bushing into the vinyl tube: Locate a 1¼” diameter by ¾” pipe bushing. Insert the pipe bushing into the opposite end of the vinyl tube that you connected in Step 2. Ensure it fits securely to maintain the integrity of the fluid pathway.

Step 4: Attach the elbow adapter to the pipe bushing: Take a ¾" mght X ⅜" hose elbow adapter. Attach the elbow adapter to the pipe bushing from Step 3. Confirm that the connection is firm, as this will facilitate the transition to the T-connector.

Step 5: Connect the elbow adapter to the T-connector: Obtain a ⅜” valve T-connector. Use a polyethylene tube to connect the elbow adapter to one of the ports on the T-connector. Make sure the connection is secure, preventing any leakage.

Step 6: Connect the T-connector to the John Guest valve: Connect the other port of the T-connector to the John Guest valve using another section of polyethylene tube. Ensure that the connections are tight, as this will help control fluid flow later.

Step 7: Connect the T-connector to polyvinyl chloride (PVC) tubing: Using a plastic hose barb reducer, connect the third port of the T-connector to a section of PVC tubing. Verify that this connection is secure to maintain proper fluid delivery.

Step 8: Connect PVC tubing to the IV fluid bag: Take the PVC tubing and attach it to an IV fluid bag using a white spike male connector. Fill the IV bag with a suitable IV fluid mixed with red food coloring to simulate blood. Ensure all connections are secure by using silicone glue and that there are no leaks throughout the system.

**Figure 2:**
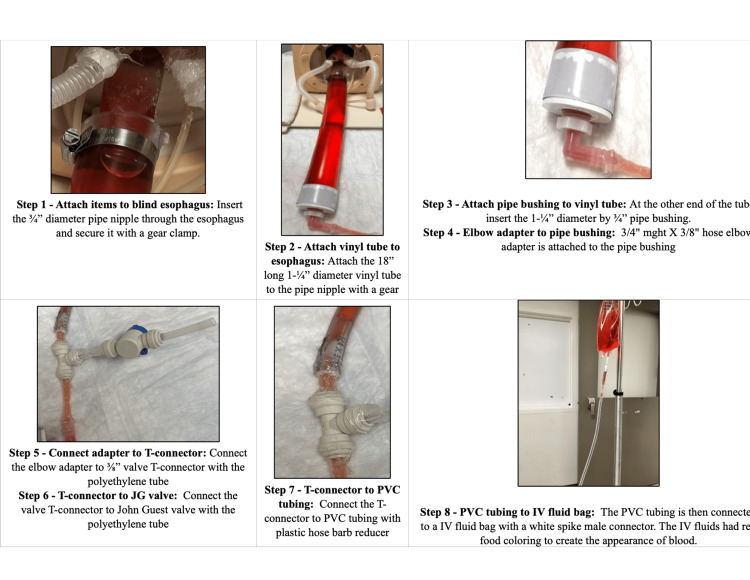
Steps for the task trainer construction

Figure 2 describes this process step-by-step with pictures.

Balloon tamponade device insertion training session

Before the session, we purchased two expired Minnesota tubes and Sengstaken-Blakemore tubes from an online medical supplier. We placed an endotracheal tube in the manikin, set up suction, and gathered all the supplies needed to insert the balloon tamponade device.

The training session included 28 emergency medicine residents of all training levels (Table 1). There were 12 postgraduate year 1 (PGY-1), 5 PGY-2, and 11 PGY-3 level residents present for this session. The residents were sent instructions to prepare for the session by reviewing the EM:RAP^Ⓡ^ video on the insertion of a balloon tamponade device [14]. Pre-session preparation included approximately 30 minutes to review the video and learn the indications and contraindications of the procedure. The learners were divided into small groups of five people, and they were given approximately 45 minutes for this station.

**Table 1:** Balloon tamponade device task trainer survey results PGY: postgraduate year

Level of learner	Number of learners	Have you ever inserted a balloon tamponade device in a patient before?	How many times have you had training in inserting a balloon tamponade device before?	Have you watched the training video for inserting a balloon tamponade device?		Time to insert balloon tamponade device in task trainer (min:sec)
				Yes	No	
PGY 1	12	0	0	8	4	3.57
PGY 2	5	0	0	5	0	3.02
							PGY 3	11	0	0	11	0	4.27
Total	28	0	0	24	4	3.49							

At the beginning of the session, the learners were asked to complete a paper survey that had both pre- and post-session questions. Appendix A contains the survey that was distributed during the session.

The training session began with a review of the indications and contraindications for balloon tamponade device placement. Learners were introduced to the key differences between the Minnesota and Sengstaken-Blakemore (SB) tubes, with a focus on the Minnesota tube, which is more commonly available at our institution. The insertion steps were demonstrated while learners followed along, guided by the EM:RAP instructional video they had watched.

For hands-on practice, learners used the Minnesota tube and were timed during the insertion process, with timing starting at the initiation of the procedure. An instructor was present at the bedside to provide guidance as needed. To facilitate the tube's insertion, mineral oil was used as a lubricant.

To simulate the clinical scenario of a massive upper gastrointestinal bleed, the clamps were opened once learners positioned themselves at the head of the bed, causing fluid to rapidly fill the tube and accumulate in the mouth, mimicking active bleeding. As in real-life cases, a suction setup was essential to maintain visibility during the procedure. The Minnesota tube was inserted adjacent to the endotracheal tube, and a swift insertion technique was emphasized to reduce the need for multiple attempts.

The insertion steps adhered to the EM:RAP video instructions, with a modification to inflate the stomach balloon with only 50 mL of air to prevent rupture of the balloon in the vinyl tube. Once the stomach balloon was inflated, the simulated bleeding in the mouth and esophagus stopped promptly. Learners then proceeded to inflate the esophageal balloon using a manometer. Following inflation, any remaining fluid in the mouth was suctioned. The session concluded with participants completing a post-training survey.

## Results

Since the survey was administered in paper format, we achieved a 100% response rate. The survey results are summarized in Table 1. All participating emergency medicine residents reported that they had never placed a balloon tamponade device in a patient before, nor had they previously undergone a training session for this procedure. While four PGY-1 residents had not seen the EM:RAP instructional video prior to the session, the remainder of the participants had.

The overall average time to insert the balloon tamponade device across all levels of training was 3 minutes and 49 seconds. When broken down by training level, PGY-1 residents averaged 3 minutes and 57 seconds, PGY-2 residents averaged 3 minutes and 2 seconds, and PGY-3 residents averaged 4 minutes and 27 seconds.

**Figure 3:**
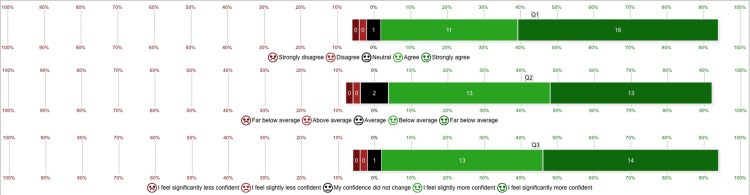
Likert scale of responses for post-survey questions Q1: The upper gastrointestinal bleed task trainer created a realistic experience in learning how to insert a balloon tamponade device. Q2: How would you rate the balloon tamponade device insertion simulation station compared to previous lectures or workshops? Q3: How did the balloon tamponade device insertion simulation affect your confidence in managing a patient with a massive upper gastrointestinal bleed?

Post-survey responses, shown in Figure 3, used a Likert scale to evaluate participants’ experiences. Sixteen learners strongly agreed, and 11 learners agreed that the task trainer provided a realistic experience for learning balloon tamponade device insertion, while one participant was neutral.

Regarding the simulation station's quality, 13 participants rated it as "far above average" compared to previous lectures or simulation experiences; another 13 rated it as "above average," and two rated it as "average."

When asked about their confidence in managing massive UGIB after the session, 14 residents reported feeling "significantly more confident," 13 felt "slightly more confident," and one participant noted no change in their confidence level.

Some of the comments we received on the survey included: “Very realistic task trainer,” "The trainer was helpful since it simulated the return of blood," and "Very realistic trainer. I feel very prepared for the real thing," "Super helpful regarding logistics & efficient procedural flow," "Realistic, easy steps to follow," "Thank you for this learning opportunity!," "Very high-fidelity," "Great experience," "Lubricant was difficult. Bougie probably made insertion easier" and "Great training of rare procedures. Now, I feel like I could at least not look really stupid.” Unfortunately, the learner who felt neutral about the realistic experience did not leave any comments.

## Discussion

The balloon tamponade device task trainer significantly enhanced the confidence of emergency medicine residents in managing massive UGIB. Despite learners having no prior training or experience with the procedure, they were asked to assess the realism of the task trainer to evaluate its usefulness as a training tool. Learners reported a highly realistic training experience, highlighting the task trainer's value in providing meaningful practice for this high-acuity, low-frequency procedure.

Participants were able to insert the tube quickly, regardless of their post-graduate year level, suggesting that trainees at all stages can become proficient with adequate preparation and training. While there is no established benchmark for the time required to place the device in clinical settings, our study aimed to measure the time taken under optimal conditions on the task trainer to offer an objective reference for learners.

Feedback indicated that the training session was superior to other educational methods, offering a practical experience essential for effective skill acquisition. Although two other balloon tamponade task trainers have been described in the literature [12,13], they are limited by a lack of realism despite being cost-effective. Our task trainer addresses this gap by simulating fluid return, providing learners with the critical feedback needed to ensure successful device placement. This model uniquely combines affordability with the realistic experience necessary for effective training.

To optimize the training experience, two procedural adjustments were made: inflating the stomach balloon to only 50 mL of air to prevent rupture within the vinyl tube and using mineral oil to facilitate tube insertion. These changes helped ensure a smoother training process while following the rest of the procedure as demonstrated in the instructional video.

Looking ahead, the task trainer's application could extend beyond balloon tamponade device placement to include training for airway management in patients with massive UGIB. Techniques such as traditional endotracheal intubation, suction-assisted laryngoscopy, airway decontamination, and intentional esophageal intubation could be practiced in this model [15]. Future studies could investigate its effectiveness in teaching these airway techniques, potentially broadening its role in emergency medicine education.

Limitations

Our study has several limitations. First, the task trainer was constructed using a manikin, which may limit its reproducibility for programs or institutions that lack access to similar equipment. Additionally, the time it took for learners to place the device may not accurately reflect real-world conditions, as the training did not include distractors or clinical stressors typically encountered in patient care. Moreover, the availability of all necessary equipment at the bedside during the training session does not simulate real-life scenarios, where setup time may vary. Finally, while this study focused on assessing the realism of the task trainer and the confidence of the learners, it did not evaluate the impact of training on clinical competency or patient outcomes.

## Conclusions

Emergency medicine residents need training in various high-acuity low-frequency procedures, including training in the placement of balloon tamponade devices in patients with massive upper gastrointestinal bleeds, so that they can be prepared to manage the patients efficiently and confidently. The materials needed to construct the task trainer are readily available at several places. The balloon tamponade device task trainer can be built in eight easy steps and is cost-efficient. Along with an online video and training session, learners were able to successfully place the Minnesota tube in a timely fashion. Learners found this task trainer to be beneficial in improving confidence in managing patients with massive UGIB. Furthermore, they also found the task trainer to be realistic and far better than other forms of education modalities.

## Figures and Tables

**Figure 4:**
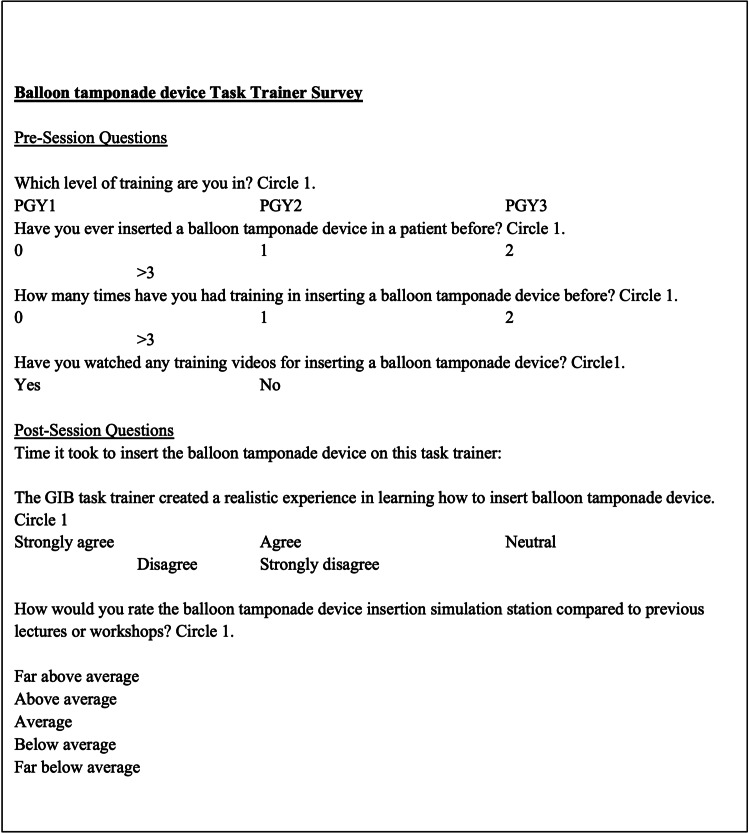
Survey distributed during the session

## Appendices

Appendix A
